# Depletion or over-expression of Sh3px1 results in dramatic changes in cell morphology

**DOI:** 10.1242/bio.013755

**Published:** 2015-10-12

**Authors:** Lawrence Hicks, Guojun Liu, Fiona P. Ukken, Sumin Lu, Kathryn E. Bollinger, Kate O'Connor-Giles, Graydon B. Gonsalvez

**Affiliations:** 1 Cellular Biology and Anatomy, Georgia Regents University, Augusta, GA 30912, USA; 2 Laboratory of Genetics, and Laboratory of Cell and Molecular Biology, University of Wisconsin-Madison, Madison, WI 53706, USA; 3 James and Jean Culver Vision Discovery Institute, Georgia Regents University, Augusta, GA 30912, USA

**Keywords:** BAR domain, Actin nucleation, Lamellipodia, Protrusion, Sorting nexin, Tubule

## Abstract

The mammalian Sorting Nexin 9 (Snx9) family consists of three paralogs: Snx9, Snx18 and Snx33. Most of the published literature to date has centered on the role of Snx9 in clathrin-mediated endocytosis (CME). Snx9 contains an Sh3 domain at its N-terminus and has been shown to interact with Dynamin and actin nucleation factors via this domain. In addition to the Sh3 domain, Snx9 also contains a C-terminal BAR domain. BAR domains are known to sense and/or induce membrane curvature. In addition to endocytosis, recent studies have implicated the Snx9 family in diverse processes such as autophagy, macropinocytosis, phagocytosis and mitosis. The Snx9 family is encoded by a single gene in *Drosophila* called *sh3px1*. In this report, we present our initial characterization of *sh3px1*. We found that depletion of Sh3px1 from *Drosophila* Schneider 2 (S2) cells resulted in defective lamellipodia formation. A similar phenotype has been reported upon depletion of Scar, the actin nucleation factor implicated in forming lamellipodia. In addition, we demonstrate that over-expression of Sh3px1 in S2 cells results in the formation of tubules as well as long protrusions. Formation of these structures required the C-terminal BAR domain as well as the adjacent Phox homology (PX) domain of Sh3px1. Furthermore, efficient protrusion formation by Sh3px1 required the actin nucleation factor Wasp. Tubules and protrusions were also generated upon over-expressing the mammalian orthologs Snx18 and Snx33 in S2 cells. By contrast, over-expressing Snx9 mostly induced long tubules.

## Abstract (precis)

**Summary:** Proteins containing BAR domains are known to generate membrane curvature. Some BAR domains generate tubules upon over-expression in cells, whereas others generate membrane protrusions. We demonstrate that Sh3px1, the *Drosophila* ortholog of the Snx9 family, is capable of inducing both tubules and protrusions.

## INTRODUCTION

Numerous cellular processes such as endocytosis, phagocytosis, vesicular sorting, autophagy, and cell migration require a relatively flat lipid bilayer to become curved (McMahon and Boucrot, 2015). Proteins containing a Bin-Amphiphysin-Rvs (BAR) domain are emerging as important players in generating membrane curvature, and as such, have been implicated in the above-mentioned cellular processes (Mim and Unger, 2012; Suetsugu and Gautreau, 2012).

In general, there are three main kinds of BAR domains; classical BAR, Fes-Cip4 homology BAR (F-BAR), and inverse BAR (I-BAR) (Mim and Unger, 2012). Over the past several years, the structures of select BAR domains have been solved. Based on these studies, it is generally accepted that BAR domains dimerize to generate either a concave or convex membrane binding surface (Masuda and Mochizuki, 2010; Qualmann et al., 2011). Proteins containing a classical BAR or F-BAR domain have basic amino acids positioned in a concave membrane binding surface (Frost et al., 2008; Peter et al., 2004; Shimada et al., 2007). Consequently, these proteins typically sense membranes with positive curvature. When incubated with purified liposomes, or when over-expressed in cells, most classical and F-BAR domain proteins generate membrane tubules (Mim and Unger, 2012). By contrast, I-BAR proteins adopt a convex membrane binding conformation upon dimerization (Millard et al., 2005; Mim and Unger, 2012). These proteins therefore bind to negatively curved membranes, and when over-expressed in cells, induce the formation of membrane protrusions (Millard et al., 2005). Thus, in a simplified view, the type of membrane deformation induced by a BAR domain protein corresponds to the shape adopted by the BAR domain dimer.

There are some exceptions to this rule. For instance, in a surprising finding, Guerrier et al., demonstrated that the F-BAR protein, srGAP2, behaves much like an I-BAR protein (Guerrier et al., 2009). Its over-expression in cells resulted in the formation of membrane protrusions (Guerrier et al., 2009). Cip4, which also contains an F-BAR domain, forms tubules in Cos7 cells, whereas its over-expression in cortical neurons results in the formation of protrusions (Itoh et al., 2005; Saengsawang et al., 2012). Over-expression of the isolated F-BAR domain of Pacsin2 in HeLa cells results in the formation of tubules and small membrane protrusions known as micro-spikes (Shimada et al., 2010). Finally, the F-BAR domain of the *Drosophila* protein Nervous wreck (Nwk) and its mammalian homolog were also shown to form protrusions when over-expressed in cells (Becalska et al., 2013). The mechanism by which these F-BAR domain proteins induce protrusion formation remains an open question.

Sorting nexins are a family of proteins that are known to function in various aspects of vesicular sorting (Cullen, 2008; Cullen and Korswagen, 2012). Consistent with this role, sorting nexins contain a membrane binding domain known as a phox-homology (PX) domain. Several of the sorting nexins also contain a classical BAR domain (Cullen, 2008; Cullen and Korswagen, 2012). In addition, the Snx9 family of sorting nexins contain an N-terminal Src-homology 3 (Sh3) domain. In mammals, the Snx9 family consists of three paralogs; Snx9, Snx18 and Snx33. Initial studies implicated a role for Snx9 in the early stages of clathrin-mediated endocytosis (Lundmark and Carlsson, 2009; Posor et al., 2013). Consistent with this function, Snx9 interacts with core endocytic factors such as Clathrin heavy chain, Dynamin, and the Adaptor protein AP2 (Lundmark and Carlsson, 2002, 2003). Recent findings have also suggested roles for the Snx9 family in diverse processes such as fluid-phase endocytosis, autophagy, macropinocytosis, phagocytosis, and mitosis (Almendinger et al., 2011; Knaevelsrud et al., 2013; Lu et al., 2011; Ma and Chircop, 2012; Wang et al., 2010; Yarar et al., 2007).

What is the mechanism by which Snx9 performs these functions? One complicating factor in answering this question stems from the fact that the Snx9 family is present as three paralogous genes in mammals, with various cell types expressing more than one paralog (Park et al., 2010). In contrast to mammals, the Snx9 family is represented by a single gene in *Drosophila melanogaster*, *sh3px1*. Our goal, therefore, is to use the genetic tools of *Drosophila*, and the simplicity afforded by a single gene, to understand the various *in vivo* functions of the Snx9 gene family. This report describes our initial characterization of Sh3px1 in *Drosophila* Schneider 2 (S2) cells. Sh3px1 displays a complex localization pattern in S2 cells, localizing to cytoplasmic foci as well as the cell cortex. Depletion of Sh3px1 compromises the ability of S2 cells to flatten and extend lamellipodia. Our results suggest that Sh3px1 might function along with the actin nucleation factor, Scar, in formation of lamellipodia. In addition, we present the surprising finding, that despite containing a classical BAR domain, Sh3px1 is capable of inducing both tubules and membrane protrusions in S2 cells. We further demonstrate that this function requires an intact PX-BAR domain. Protrusion formation by Sh3px1 also appears to require the actin nucleation factor, Wasp.

## RESULTS

### Localization of endogenous Sh3px1 in *Drosophila* S2 cells

**Fig. 1.**
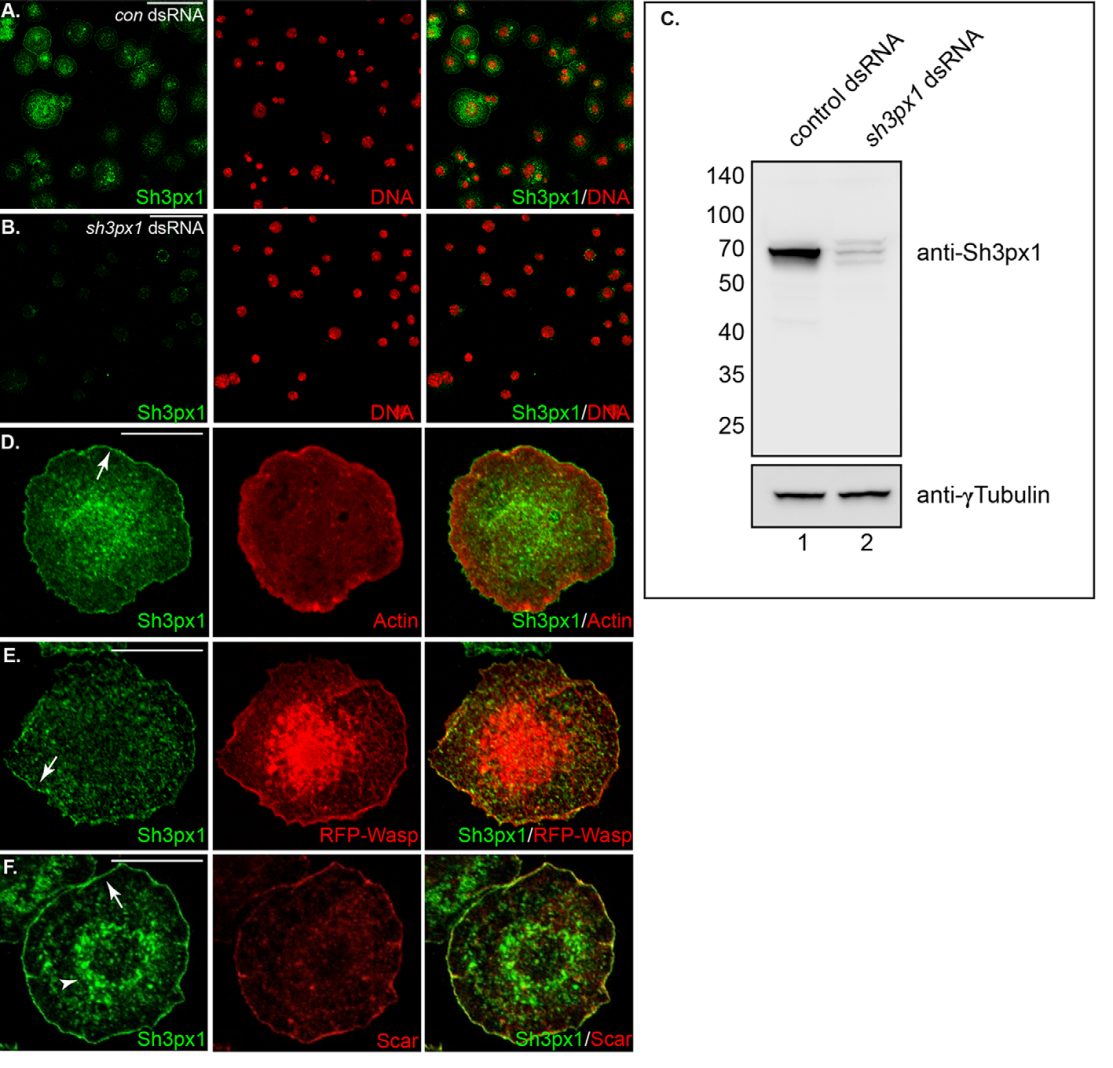
**Localization of endogenous Sh3px1.** (A,B) *Drosophila* S2 cells were treated with dsRNAs against *gfp* (A) or *sh3px1* (B). Four days after dsRNA treatment, the cells were spotted onto concanavalin A (con A) coated coverslips and allowed to adhere for 2 h. The cells were then fixed and analyzed using an antibody against Sh3px1 (green). The cells were also counterstained with DAPI to reveal nuclei (red). (C) S2 cells were treated with dsRNAs against *gfp* (lane 1) or *sh3px1* (lane 2). Lysates were prepared from these cells and run on an SDS-PAGE gel. The proteins were transferred to nitrocellulose and processed for western blot analysis using the indicated antibodies. (D) S2 cells were spotted onto con A coverslips and allowed to adhere for 2 h. The cells were then fixed and processed for immunofluorescence using an antibody against Sh3px1 (green). The cells were also counterstained with TRITC conjugated Phalloidin to reveal F-actin (red). Sh3px1 localized adjacent to the cortical actin network (arrow) as well as to internal foci. (E) S2 cells were transfected with a plasmid expressing RFP-tagged Wasp. Four days after transfection, the cells were spotted onto Con A coverslips and allowed to adhere for 2 h. The cells were then fixed and processed for immunofluorescence using an antibody against Sh3px1 (green). Sh3px1 co-localized with RFP-Wasp at the cell cortex (arrow). (F) S2 cells were spotted onto con A coverslips and allowed to adhere for 2 h. The cells were then fixed and processed for immunofluorescence using an antibody against Sh3px1 (green) and Scar (red). Sh3px1 co-localized with endogenous Scar at the cell cortex (arrow). In addition, a perinuclear enrichment of Sh3px1 could be detected in approximately 45% of cells (arrowhead). Scale bars: 15 μm.

In order to begin our analysis of Sh3px1, we generated a polyclonal antibody against full-length Sh3px1. The rabbit serum was purified against recombinant Sh3px1 and tested for activity and specificity. *Drosophila* Schneider 2 (S2) cells that were treated with either a control dsRNA or with dsRNA against *sh3px1* were spotted onto concanavalin A (con A) coated coverslips. Con A coating is required for the normally semi-adherent S2 cells to attach firmly to coverslips (Rogers and Rogers, 2008). The cells were fixed and processed for immunofluorescence using the Sh3px1 antibody. Abundant signal could be detected with control cells, but not with cells treated with dsRNA against *sh3px1* (Fig. 1A,B). As a further test, lysates were prepared from S2 cells treated with a control dsRNA or with dsRNA against *sh3px1*. The lysates were run on a gel and analyzed by western blotting. As expected, the antibody detected a single band of approximately 70 kD in control lysates (Fig. 1C, lane 1). The intensity of this band was significantly reduced in the dsRNA treated sample (Fig. 1C, lane 2). We therefore conclude that S2 cells express Sh3px1, and that the antibody is capable of detecting the protein in a native and denatured form.

We next examined the intracellular localization of Sh3px1. Upon attaching to con A coated coverslips, S2 cells flatten and extend circumferential lamellipodia (Rogers et al., 2003). The net result is a cell with a centrally placed nucleus and a cortical band of filamentous actin (F-actin) that surrounds the entire cell (Rogers et al., 2003). Electron microscopy studies have demonstrated that the cortical band consists of dendritically branched actin filaments (Biyasheva et al., 2004). Sh3px1, as well as its human ortholog Snx9, have been shown to associate with nucleators of actin filament formation such as Wasp and the Apr2/3 complex (Badour et al., 2007; Shin et al., 2008; Worby et al., 2001). Consistent with these reported interactions, Sh3px1 was enriched at the cell cortex, adjacent to the lamellipodia (Fig. 1D). Interestingly, Sh3px1 was localized more distal to the cortical F-actin band (Fig. 1D). In addition, Sh3px1 partially co-localized with RFP-tagged Wasp and endogenous Scar at the cell cortex (Fig. 1E,F, arrow). Diffusely distributed puncta of Sh3px1 were also observed in most cells (Fig. 1D-F). Furthermore, approximately 45% of cells, displayed a peri-nuclear enrichment of Sh3px1 (Fig. 1F, arrowhead; *n*=100).

### Sh3px1 is required for lamellipodia formation

**Fig. 2.**
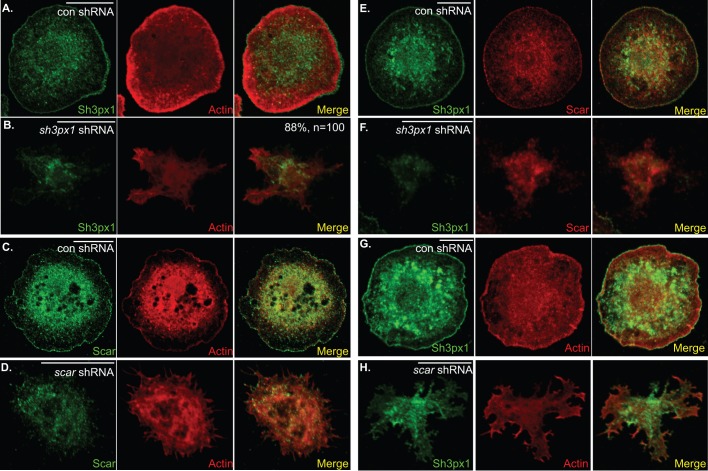
**Depletion of Sh3px1 results in defective lamellipodia formation.** (A,B) S2 cells were transfected with a plasmid expressing a control shRNA targeting the yeast *gal80* gene (A) or with a plasmid expressing shRNA targeting *sh3px1* (B). The cells were also transfected with the Act5c-Gal4 plasmid in order to induce expression of the shRNA. Three days after transfection, the cells were spotted onto con A coverslips and allowed to adhere for 2 h. The cells were then fixed and processed for immunofluorescence using an antibody against Sh3px1 (green). The cells were also counterstained to reveal F-actin (red). The percentage of Sh3px1 depleted cells displaying the lamellipodia defect is indicated in panel B. (C,D) S2 cells were transfected with a plasmid expressing the *gal80* control shRNA (C) or with a plasmid expressing an shRNA targeting *scar* (D). Three days after transfection, the cells were fixed, processed using an antibody against Scar (green), and were counterstained to reveal F-actin (red). (E,F) S2 cells were transfected with a plasmid expressing the *gal80* control shRNA (E) or with a plasmid expressing an shRNA targeting *sh3px1* (F). Three days post transfection, the cells were fixed and processed using antibodies against Scar (red) and Sh3px1 (green). (G,H) S2 cells were transfected with a plasmid expressing the *gal80* control shRNA (G) or with a plasmid expressing an shRNA targeting *scar* (H). Three days later, the cells were fixed and processed using an antibody against Sh3px1 (green). The cells were also counterstained to reveal F-actin (red). Scale bars: 15 μm.

In order to determine the *in vivo* function of Sh3px1, S2 cells were transfected with either a control short hairpin RNA (shRNA) or an shRNA targeting *sh3px1*. The control shRNA was designed against a yeast gene, and as such, the targeting sequence is not present in the *Drosophila* genome. Cells expressing the control shRNA displayed the normal round morphology with a prominent cortical band of actin, indicative of lamellipodia (Fig. 2A). By contrast, the morphology of cells expressing the shRNA against *sh3px1* was significantly altered (Fig. 2B, Fig. S1A). The Sh3px1-depleted cells were able to attach to the coverslips. However, spreading of cells and lamellipodia formation were defective. As such, the cortical band of actin was not observed (Fig. 2B).

A similar phenotype has been observed in cells depleted of Scar, the actin nucleating factor that functions in lamellipodia formation (Biyasheva et al., 2004; Kunda et al., 2003; Rogers et al., 2003). Our results are consistent with these previous reports (Fig. 2C,D). Collectively, these observations suggest that Sh3px1 might function together with Scar in forming lamellipodia. Consistent with this notion, the cortical localization of Scar was lost in cells depleted of Sh3px1 (Fig. 2E,F). Similarly, the cortical localization of Sh3px1 was disrupted in Scar-depleted cells (Fig. 2G,H).

### Formation of tubules and protrusions upon over-expression of Sh3px1

The domain organization of Sh3px1 is shared by its mammalian orthologs: Snx9, Snx18 and Snx33. As suggested by its name, Sh3px1 contains a Src-homology 3 (Sh3) domain as well as a phox-homolgy (PX) domain (Fig. 4A). In addition, Sh3px1 also contains a C-terminal BAR domain (Fig. 4A). BAR domains are known for sensing/inducing curvature in membranes (Mim and Unger, 2012). Pylypenko et al., have solved the structure of the Snx9 PX-BAR domain (Pylypenko et al., 2007). The PX-BAR of Snx9 dimerizes and adopts a concave conformation typically seen with classical BAR domain proteins (Pylypenko et al., 2007).

A commonly used strategy for examining the membrane shaping properties of BAR domain proteins is to over-express the protein in cells. For instance, over-expression of Snx9 in HeLa and Cos7 cells results in formation of long membrane tubules (Pylypenko et al., 2007; Shin et al., 2008). A similar phenotype is observed upon over-expressing other BAR domain proteins such as Amphiphysin and Endophilin 3, and F-BAR proteins such as FBP17 and CIP4 (Itoh et al., 2005; Lee et al., 2002). By contrast, over-expression of inverse BAR (I-BAR) domain proteins result in cell protrusions (Mattila et al., 2007; Yamagishi et al., 2004; Zhao et al., 2011).

**Fig. 3.**
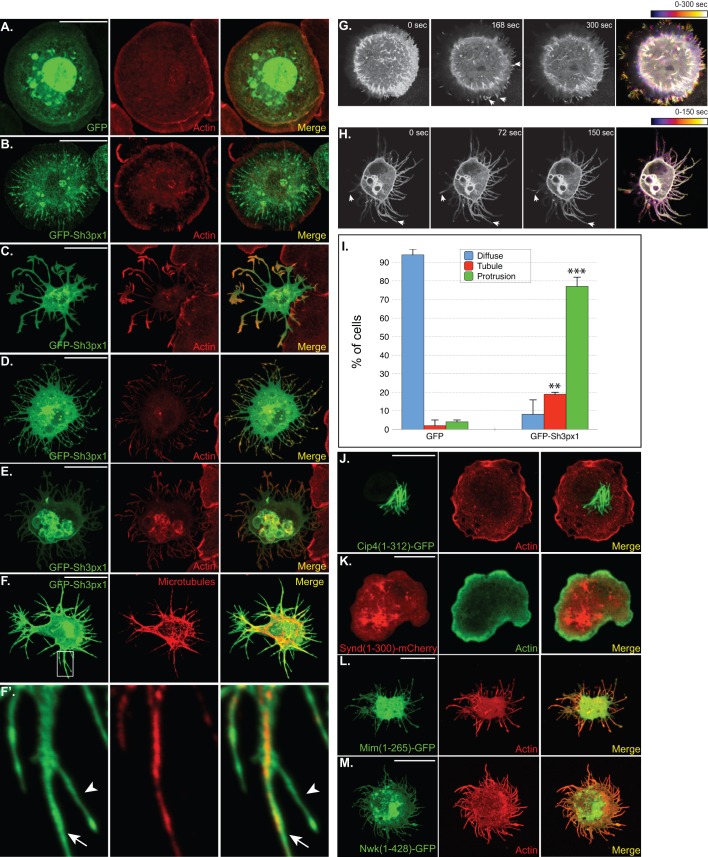
**Formation of tubules and protrusions by GFP-Sh3px1.** (A-E) S2 cells were transfected with a plasmid encoding GFP (A) or GFP-Sh3px1 (B-E) and the Act5c-Gal4 plasmid that was required for driving expression of the fusion proteins. Two days after transfection, the cells were spotted onto con A coverslips and allowed to adhere for 2 h. The cells were then fixed, counterstained with Phalloidin-TRITC, and imaged. (F) S2 cells were transfected with a plasmid encoding GFP-Sh3px1 and spotted onto con A coverslips as noted above. The cells were then fixed in methanol at −20°C and stained with an antibody against Alpha-tubulin to reveal the microtubule network (red). Panel F′ represents a magnified view of the region outlined by the rectangle in F. (G) S2 cells were transfected with a plasmid encoding GFP-Sh3px1. Two days after transfection, the cells were spotted onto con A coated glass bottom dishes and allowed to adhere. The cells were then imaged live. Select time points (0 s, 168 s, and 300 s) from one such imaging experiment are shown. The panel on the far right represents a temporal color coded image. Images at the start of the experiment are depicted in purple and those at the end of the experiment are shown in white. The arrows indicate accumulation of GFP-Sh3px1 in small membrane protrusions that continue to elongate during the course of the experiment. (H) Similar treatment as panel G. Select time points (0 s, 72 s, and 150 s) from a live imaging experiment are shown here. The panel on the far right represents a temporal color-coded image. Arrows indicate membrane protrusions that continue to elongate during the time course of the experiment. Scale bars: 15 μm. (I) Quantification of the phenotypes observed in panels A-E. Cells in which no particular localization pattern was detected were counted as ‘Diffuse’. Cells in which membrane protrusions were observed were scored as ‘Protrusions’. Cells containing tubular structures were scored as ‘Tubules’. The graph represents data from three independent experiments. For each experiment, 100 cells were counted for each construct. The error bars represent standard deviation. **P=0.0007, ***P≤0.0001, Unpaired *t*-test. (J-M) S2 cells were transfected with the following expression constructs, Cip4-GFP (J), Syndapin-mCherry (K), Mim-GFP (L), and Nwk-GFP (M). Two days after transfection, the cells were spotted onto con A coated coverslips and allowed to adhere. The cells were then fixed, counterstained to reveal F-actin, and imaged.

In order to determine whether Sh3px1 is capable of inducing membrane curvature, we over-expressed full-length GFP-Sh3px1 in S2 cells. Consistent with the properties of its mammalian ortholog, tubules decorated with GFP-Sh3px1 could be detected in over-expressing cells (Fig. 3B,I). Tubular structures were rarely observed in cells over-expressing GFP alone (Fig. 3A,I). Tubules generated by Snx9 are known to arise from the plasma membrane (Shin et al., 2008). We therefore attempted to determine whether this was also true of tubules generated by GFP-Sh3px1. Cells over-expressing GFP-Sh3px1 were labeled with numerous membrane dyes such as FM4-64, DiI, wheat germ agglutinin, and Cell Mask. Unfortunately, these reagents labeled internal membraneous structures in S2 cells and convincing plasma membrane labeling was not observed. Thus, at this time, it is unclear whether the tubules generated by GFP-Sh3px1 are originating from the plasma membrane.

Surprisingly, however, the tubule phenotype was only observed in approximately 15% of GFP-Sh3px1 over-expressing cells (Fig. 3I). The majority of these cells displayed long protrusions (Fig. 3C,D,E,I). Within this group, a few variations were observed. Most protrusions were branched (Fig. 3C). Occasionally, protrusions containing bulges or nodules were observed (Fig. 3D). A small number of cells also displayed internal vesicular structures that were positive for both GFP-Sh3px1 and actin in addition to long protrusions (Fig. 3E). The reason for this phenotypic variation is unclear. For purposes of quantification, all three groups depicted in panels C, D and E of Fig. 3 were scored as cells containing ‘protrusions’. Similar results were obtained by over-expressing an untagged version of Sh3px1 (Fig. S2A,B). To the best of our knowledge, this is the first description of a classical BAR domain protein that is capable of inducing both tubules and protrusions.

The actin cytoskeleton has been implicated in formation of tubules and protrusions by BAR domain proteins (Itoh et al., 2005). We therefore examined F-actin in cells over-expressing GFP-Sh3px1. As noted previously, untransfected cells or cells over-expressing GFP, attach to con A coated coverslips, flatten and form lamellipodia (Fig. S2C; Fig. 3A). Thus, control cells display a strong cortical band of F-actin. The localization of F-actin was relatively unchanged in GFP-Sh3px1 over-expressing cells that displayed the tubule phenotype (Fig. 3B). However, in GFP-Sh3px1 over-expressing cells that formed protrusions, the cortical band of actin was not observed and most of the signal for F-actin was observed in the tips of the protrusion (Fig. 3C,D,E; Fig. S2C).

In order to determine whether the protrusions represent very long lamellipodia or filopodia, cells over-expressing RFP-Sh3px1 were fixed and stained using antibodies against Scar and Enabled (Ena). Scar has been used as a marker for lamellipodia and Ena has been used to mark filopodia (Biyasheva et al., 2004). Scar and Ena were not excluded from the protrusions induced by Sh3px1 over-expression. However, unlike F-actin, they were not specifically enriched at the tips of the protrusions (Fig. S3A,B). We therefore propose that these structures are neither purely lamellipodia nor filopodia, but rather a neomorphic actin-rich structure produced as a consequence of Sh3px1 over-expression.

We next determined whether microtubules were also present within GFP-Sh3px1 induced protrusion. Fixed cells were stained using an antibody against Alpha-tubulin. Many, but not all of the protrusions, contained microtubules (Fig. 3F, red). The long protrusions usually contained microtubules, but the shorter branches were often negative for Alpha-tubulin staining (Fig. 3F′, arrow vs arrowhead). One interpretation of this result is that microtubules are not the driving force behind protrusion formation. Rather, it is likely that microtubules gradually grow into the protrusions once they are formed.

Tubules generated by BAR domain proteins and protrusions formed by I-BAR proteins are dynamic (Guerrier et al., 2009; Shin et al., 2008). We therefore examined whether this was also true of the membrane deformations induced by Sh3px1. S2 cells that over-expressed either GFP or GFP-Sh3px1 were imaged live (Movies 1, 2, 3). As noted upon imaging fixed cells, GFP over-expressing cells did not form tubules or protrusions (Movie 3). By contrast, both types of membrane deformations were observed with GFP-Sh3px1 over-expressing cells. Fig. 3G depicts a time series of a cell over-expressing GFP-Sh3px1 (Movie 1). At the start of the imaging experiment, several tubules are visible within the cell. Within three minutes, we observed the formation of small protrusions at the cell periphery. Importantly, GFP-Sh3px1 was enriched within these protrusions (Fig. 3G, 168 s, arrows). At the end of the five-minute imaging experiment, the cell displayed both tubular structures and protrusions (Fig. 3G, 300 s). Thus, not only can GFP-Sh3px1 induce the formation of tubules and protrusions; this can even happen in the same cell. The panel on the right represents a temporal color-coded image, where the images at the start of the experiment are depicted in blue and those at the end of the experiment are shown in white. Fig. 3H depicts a similar time series of a cell over-expressing GFP-Sh3px1 (Movie 2). This cell has already formed long protrusions. However, live imaging reveals that even these longer protrusions are dynamic (Fig. 3H, arrows). As before, the panel on the right displays a temporal color-coded image.

Our results thus far demonstrate that GFP-Sh3px1 is able to form tubules and protrusions in S2 cells. Although this was not observed in GFP over-expressing cells, we wished to further test the specificity of this phenotype. Consistent with published results, over-expressing the F-BAR domain of Cip4 formed long tubular structures (Fig. 3J) (Becalska et al., 2013; Fricke et al., 2009). Over-expressing the F-BAR domain of Syndapin formed internal membrane clusters (Fig. 3K). A similar phenotype was also observed by Becalska et al. (2013). Syndapin is the *Drosophila* ortholog of mammalian Pacsin. As expected, over-expressing the I-BAR domain of Missing in metastasis (Mim) resulted in long protrusions (Fig. 3L) (Becalska et al., 2013). Similarly, consistent with published results, over-expressing the F-BAR domain of Nervous wreck (Nwk) also induced the formation of long protrusions (Fig. 3M) (Becalska et al., 2013). Tubular structures were not observed in cells over-expressing Mim or Nwk (data not shown). Based on these results we conclude that S2 cells do not spontaneously form tubules and protrusions. Additionally, Sh3px1 appears unique in its ability to form these seemingly opposing structures.

### The PX-BAR domain is required for tubule and protrusion formation

**Fig. 4.**
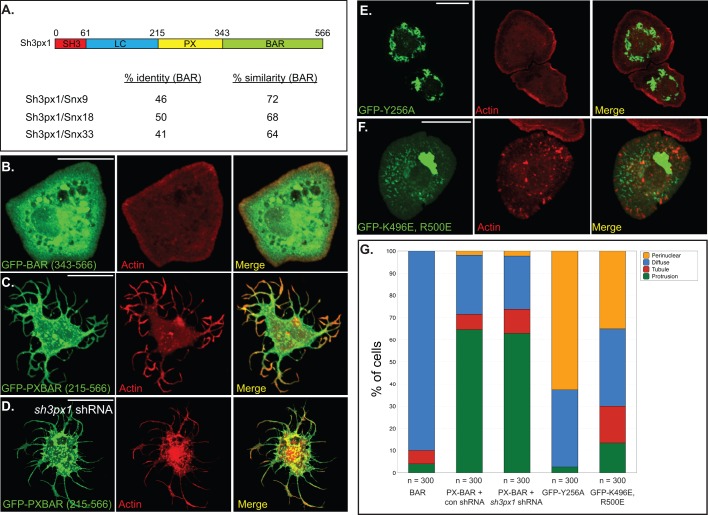
**The PX-BAR domain is required for tubule and protrusion formation.** (A) Schematic of the domain organization of Sh3px1. The amino acid residues that comprise the domains are listed. Also shown is a comparison of the percent identity and similarity in the BAR domains between Sh3px1 and Snx9, Snx18 and Snx33. (B) S2 cells were transfected with a GFP-BAR construct. Two days after transfection, the cells were spotted onto con A coverslips, allowed to adhere, fixed and counterstained to reveal F-actin (red). (C,D) S2 cells were co-transfected with a GFP-PX-BAR construct and a control shRNA (C) or an shRNA targeting endogenous *sh3px1* (D). Three days after transfection, the cells were treated as in panel B. 64.7% (±8) cells expressing the control shRNA formed protrusions in comparison to 63% (±3) for cells expressing shRNA targeting *sh3px1*. This difference is not statistically significant (*P*=0.7477). (E,F) S2 cells were transfected with GFP-PX-BAR mutant Y256A (E) or with GFP-PX-BAR mutant K496E, R500E (F). Two days after transfection, the cells were treated as in panel B. Scale bars: 15 μm. (G) Cells from the experiments represented in panels B-F were quantified. As before, cells in which GFP-Sh3px1 displayed no particular localization pattern were counted as ‘Diffuse’. Cells in which membrane protrusions were observed were scored as ‘Protrusions’. Cells containing tubular structures were scored as ‘Tubules’. Cells containing a strong enrichment of GFP signal close to the cytoplasmic face of the nuclear envelope were scored as ‘Perinuclear’. For each experiment, 100 cells were counted for each construct. The experiment was done three times.

Sh3px1 contains four recognizable domains; Sh3, Low complexity, PX, and BAR domain (Fig. 4A). As noted previously, this overall organization is shared by its mammalian orthologs. The greatest levels of identity and similarity between Sh3px1 and its mammalian orthologs lie within the Sh3, PX and BAR domains (Fig. 4A, and data not shown). We next attempted to define the critical domains and residues required for tubule and protrusion formation.

The first construct we examined was one containing the minimal BAR domain of Sh3px1 fused to GFP. The BAR domain of Sh3px1 was not capable of forming either tubules or protrusions (Fig. 4B,G). S2 cells over-expressing the minimal Sh3px1 BAR domain were able to attach and form lamellipodia, as indicated by the cortical band of F-actin (Fig. 4B).

By contrast, the PX-BAR domain of Sh3px1 fused to GFP was able to form protrusions and tubules, much like the full-length protein (Fig. 4C,G). S2 cells express endogenous Sh3px1 (Fig. 1A), and BAR domains are known to dimerize. It was therefore possible that the PX-BAR domain construct was dimerizing with endogenous Sh3px1 and forming protrusions and tubules via the activity of endogenous Sh3px1. In order address this point, we depleted endogenous Sh3px1 using an shRNA. Importantly, the sequence targeted by the shRNA is present in the full-length protein but not within the PX-BAR construct (materials and methods for details). Cells co-expressing the PX-BAR construct along with a control shRNA or with an shRNA targeting *sh3px1* formed protrusions and tubules with similar frequency (Fig. 4D,G). Thus, the PX-BAR domain of Sh3px1 appears to be necessary and sufficient to form these structures in S2 cells. A potential limitation of this conclusion is that the shRNA does not completely eliminate endogenous Sh3px1 (Fig. 2B). The residual Sh3px1 that persists might dimerize with the PX-BAR construct to generate tubules and protrusions.

With regard to Snx9, tyrosine residue 287 lies within the phosphoinositide binding pocket of the PX domain (Pylypenko et al., 2007). Mutation of this residue to alanine results in a protein that is unable to form membrane tubules when over-expressed in cells (Pylypenko et al., 2007). In order to test the importance of the PX domain in membrane deformation, we introduced a similar mutation in the corresponding tyrosine residue of Sh3px1 (Y256A). As predicted from studies of the human protein, PX-BAR (Y256A) was not able to efficiently form tubules or protrusions (Fig. 4G). Rather, this protein was either diffusely distributed throughout the cell or accumulated in a peri-nuclear region (Fig. 4E,G).

We next examined mutants within the BAR domain. The Snx9 dimer adopts a curved conformation with positively charged residues positioned on the concave membrane binding surface (Pylypenko et al., 2007). Within the human protein, residues K522 and K528 appear to be important for tubule formation. When these residues are mutated to aspartic acid, the mutant protein is no longer able to form tubules in cells, but still retains the capacity to bind membranes (Pylypenko et al., 2007). We introduced comparable mutations into Sh3px1 (K496E, R500E). For simplicity, we will refer to this mutant as PX-BAR-mut. As predicted, PX-BAR-mut had a reduced ability to form tubules and protrusion in comparison to wild-type (Fig. 4G). However, it was not completely compromised and cells containing small tubules and protrusions were observed (Fig. 4F,G; Protrusion formation in BAR alone versus PX-BAR-mut, *P*<0.0001; Tubule formation in BAR alone versus PX-BAR-mut, *P*<0.0001). As with the PX mutant, PX-BAR-mut also often accumulated in a peri-nuclear region (Fig. 4F,G).

Full-length Snx9 has been shown to co-localize with numerous vesicular structures in mammalian cells including the Golgi (Soulet et al., 2005). We therefore examined whether the peri-nuclear enrichment of PX-BAR (Y256A) and PX-BAR-mut corresponded to Golgi. Both constructs localized adjacent to Golgi vesicles, but rarely co-localized (data not shown). Similarly, minimal co-localization was observed between these constructs and markers for lysosomes and mitochondria (data not shown). At present, it is unclear what structure or organelle PX-BAR (Y256A) and PX-BAR-mut is localizing to.

### Over-expression of mammalian sorting nexins in S2 cells

As noted previously, over-expression of Snx9 in mammalian cells results in the formation of long tubules (Pylypenko et al., 2007; Shin et al., 2008). Cell protrusions were not observed. Why then does the *Drosophila* ortholog, Sh3px1, induce the formation of protrusions? One explanation is that this property is unique to Sh3px1. Alternatively, S2 cells might be more conducive to protrusion formation than HeLa or Cos7 cells. For instance, as noted previously, Cip4 forms tubules in Cos7 cells and protrusions in cortical neurons (Saengsawang et al., 2012). In order to distinguish between these possibilities, we over-expressed GFP-tagged versions of the three mammalian Snx9 family members in S2 cells.

**Fig. 5.**
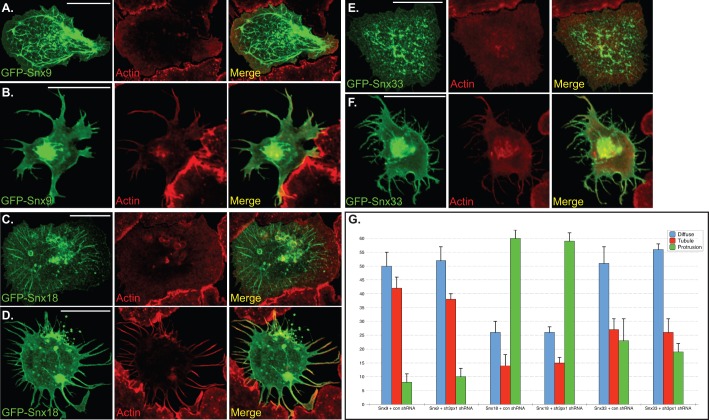
**Localization of Snx9, Snx18 and Snx33 in S2 cells.** (A,B) S2 cells were transfected with a plasmid encoding GFP-Snx9. Two days after transfection, the cells were spotted onto con A coverslips and allowed to adhere. The cells were then fixed and counterstained with Phalloidin to visualize F-actin (red). Panel A represents an example of a tubule containing cell and panel B represent a cell that has formed protrusions. (C,D) S2 cells were transfected with a plasmid encoding GFP-Snx18. The cells were treated as in the above panels. C is an example of a GFP-Snx18 cell that has formed tubules and panel D is an example of a cell with long protrusions. (E,F) S2 cells were transfected with a plasmid encoding GFP-Snx33 and treated as above. E is an example of a tubule containing cell and panel F is an example of a cell that has formed protrusions. Scale bars: 15 μm. (G) Quantification of phenotypes observed upon over-expressing GFP-Snx9, Sxn18 and Snx33. The quantification criteria for determining Diffuse, Tubule or Protrusion was the same as previously described. The graph represents data from three independent experiments. For each experiment, 100 cells were counted per construct. The error bars represent standard deviation.

Consistent with published findings in mammalian cells (Pylypenko et al., 2007; Shin et al., 2008), cells over-expressing GFP-Snx9 formed tubules (Fig. 5A,G). However, a small percentage of GFP-Snx9 over-expressing cells also formed protrusions (Fig. 5B,G). The protrusions that were formed upon GFP-Snx9 over-expression were typically shorter than those formed upon over-expressing GFP-Sh3px1 (Fig. 5B vs Fig. 3C).

Snx18 produced a phenotype that was most similar to Sh3px1, with approximately 60% of cells forming long protrusions and 15% of cells forming tubules (Fig. 5C,D,G). Snx33 was also capable of forming protrusions and tubules (Fig. 5G). However, the tubules formed upon over-expressing GFP-Snx33 were often shorter than those formed upon over-expressing GFP-Snx9 (Fig. 5E vs Fig. 5A). In addition, protrusions induced by GFP-Snx33 over-expression were shorter than those induced by over-expressing GFP-Snx18 (Fig. 5F vs Fig. 5D).

In order to determine whether these phenotypes were independent of endogenous Sh3px1, cells were co-transfected with plasmids encoding GFP-tagged versions of the three sorting nexins and an shRNA targeting *sh3px1*. This analysis revealed that depletion of Sh3px1 did not significantly alter the ability of Snx9, Snx18 or Snx33 to induce tubules and/or protrusions in S2 cell (Fig. 5G). As noted previously, a limitation of this experiment is that we cannot completely eliminate endogenous Sh3px1. The residual Sh3px1 might cooperate with its mammalian orthologs to induce these membrane deformations.

**Fig. 6.**
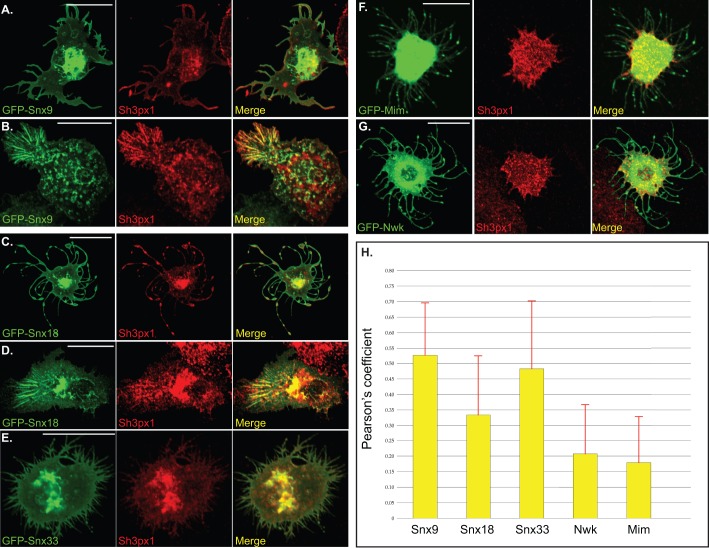
**Co-localization between endogenous Sh3px1 and BAR domain constructs.** (A-G) S2 cells were transfected with GFP-Snx9 (A,B), GFP-Snx18 (C,D), GFP-Snx33 (E), GFP-Mim (F) or GFP-Nwk (G). Two days after the transfection, the cells were fixed and processed for immunofluorescence using an antibody against endogenous Sh3px1 (red). Scale bars: 15 μm. (H) The Pearson's co-localization coefficient was quantified for endogenous Sh3px1 and the indicated GFP-tagged constructs. For each construct, 25 cells were imaged and quantified. The co-localization was quantified for the entire cell rather than using a specific region within the cell. The error bars represent standard deviation. The *P* values for comparing the level of co-localization observed between the indicated GFP-tagged constructs and endogenous Sh3px1 are shown in Fig. S4.

Previous studies suggest that only highly similar BAR domains are able to decorate the same membrane deformation (Becalska et al., 2013). For instance, a protein with a classical BAR domain and a protein with an F-BAR domain will typically not be able to decorate the same tubule due to distinct membrane sensing properties of their BAR domains. Although the structure of the PX-BAR domain of Sh3px1 has not been solved, the prediction program Phyre2 suggests that it folds into a very similar structure as Snx9 (Kelley and Sternberg, 2009). In order to test this prediction, we examined the localization of endogenous Sh3px1 in S2 cells over-expressing GFP tagged versions of the three mammalian sorting nexins. Most tubules and protrusions formed by the mammalian sorting nexins also contained endogenous Sh3px1 (Fig. 6A-E). These results suggest that the BAR domain of Sh3px1 very likely folds into a similar conformation as its mammalian orthologs. By contrast, the tubules formed upon Cip4 over-expression did not contain endogenous Sh3px1 (data not shown). Similarly, endogenous Sh3px1 was not found in the protrusions induced by GFP-Mim or GFP-Nwk over-expression (Fig. 6F,G). The Pearson's co-localization coefficient between the GFP-tagged proteins and endogenous Sh3px1 is shown in Fig. 6H.

### The role of Actin in protrusion formation

As noted previously, F-actin is localized at the tips of protrusions formed by GFP-Sh3px1 over-expression (Fig. 3C; Fig. S2C). This finding is not surprising given the numerous links between Sh3px1, Snx9, and the actin cytoskeleton. We have shown in this report that Sh3px1 is required for spreading of S2 cells and formation of lamellipodia (Fig. 2A,B). Sh3px1 as well as Snx9 have been shown to interact with Wasp (Badour et al., 2007; Worby et al., 2001). Wasp is a regulator of the Arp2/3 complex, which in turn stimulates the formation of F-actin. In addition, Snx9 has also been shown to bind directly to the Arp2/3 complex (Shin et al., 2008). Lastly, Snx9 is required for dorsal ruffle formation and for actin-dependent fluid phase endocytosis (Yarar et al., 2007). Apart from these interactions, the process of protrusion formation by I-BAR and F-BAR proteins also appears to require actin (Suetsugu and Gautreau, 2012).

**Fig. 7.**
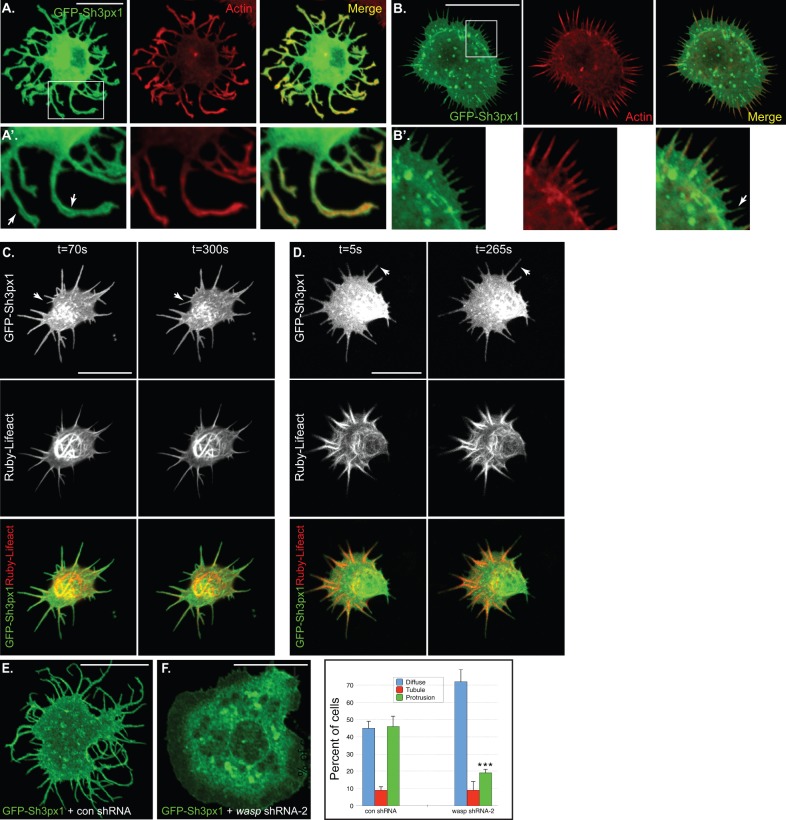
**The potential involvement of F-actin in protrusion formation.** (A,B) S2 cells were transfected with a plasmid encoding GFP-Sh3px1. Two days after transfection, the cells were spotted onto con A coverslips, allowed to adhere, and then fixed. Next, the cells were counterstained with Phalloidin-TRITC (red) to reveal F-actin. Panel A depicts a cell with long protrusions and panel B depicts a cell that is just starting to form protrusions. A′ and B′ represent magnified views of the regions outlined by rectangles in A and B, respectively. The arrows in A′ indicate that signal for Sh3px1 could be detected on the membrane of the long protrusions, whereas F-actin was observed more internally. The arrow in B′ indicates a short protrusion that contains more signal for Sh3px1 than F-actin. (C,D) Panels C and D represent select time frames from two independent live imaging experiments. S2 cells were co-transfected with plasmids encoding GFP-Sh3px1 (green) and mRuby2-Lifeact (red). Individual and merged images are shown. The arrows indicate protrusions that form and elongate during the course of the experiment. (E,F) S2 cells were co-transfected with the following combination of plasmids; GFP-Sh3px1 and the *gal80* control shRNA (E) and GFP-Sh3px1 and *wasp* shRNA-2 (F). Three days after transfection, the cells were spotted onto con A coverslips, allowed to adhere, and then fixed. Scale bars: 15 μm. (G) Quantification of phenotypes from the experiment depicted in panels E and F. The quantification criteria for determining Diffuse, Tubule, and Protrusion were as described previously. The graph represents data from three independent experiments. For each experiment, 100 cells were counted per construct. The error bars represent standard deviation. ***P=0.0018, unpaired *t*-test.

In the case of F-BAR proteins that induce protrusions, the model proposed by Becalska et al., and Shimada et al., suggests that the F-BAR protein initially generates a small inward tubule or invagination. Subsequent recruitment of actin nucleators to this site results in filament assembly and outward growth of a protrusion (Becalska et al., 2013; Shimada et al., 2010). We envisioned that a similar mechanism might also account for protrusion formation by Sh3px1. According to this model, the main role of Sh3px1 is to generate the small inward tubule or invagination. Thus, the expectation is that GFP-Sh3px1 would be enriched at the base of the protrusion. Unexpectedly, this was not what we observed. In fact, GFP-Sh3px1 was detected on the membrane of the protrusion along its entire length (Fig. 7A,A′, arrows).

Another prediction of the model is that F-actin would be observed in all protrusions. This is indeed the case for cells containing long protrusions. However, closer examination of cells that were just starting to form protrusions revealed a subtle difference. Whereas all protrusions contained signal for GFP-Sh3px1, F-actin was missing from the tips of some of the smaller protrusions (Fig. 7B,B′, arrow). In this experiment, F-actin was visualized using fluorescent Phalloidin. A caveat of this interpretation is that there might exist a population of F-actin at the tips of these smaller protrusions that is not efficiently labeled with Phalloidin.

In order to independently examine the role of actin in protrusion formation, we examined live cells that co-expressed GFP-Sh3px1 and mRuby2-Lifeact. Lifeact corresponds to a 17 amino acid peptide that binds to F-actin in live cells without affecting actin dynamics (Riedl et al., 2008). Fig. 7C and D correspond to time frames from two different cells co-expressing GFP-Sh3px1 and mRuby2-Lifeact (Movies 4, 5). As noted under fixed conditions, GFP-Sh3px1 was present in all protrusions (Fig. 7C,D). During the course of this experiments, we observed the formation of new protrusions. Interestingly, whereas the new protrusions contained GFP-Sh3px1, they were negative for mRuby2-Lifeact (Fig. 7C,D, arrows). As with Phalloidin, a limitation of this experiment is that F-actin might indeed be present within these protrusions but not efficiently bound by Lifeact (Munsie et al., 2009).

We next examined whether protrusion formation by Sh3px1 required Wasp. As mentioned previously, Wasp is a known interacting partner of Sh3px1 (Worby et al., 2001), and functions in the nucleation of actin filaments via the Arp2/3 complex. Cells were transfected with GFP-Sh3xp1 as well as a control shRNA or an shRNA targeting *wasp*. Two different shRNAs were designed against *wasp*. *wasp* shRNA-2 was found to be more effective at reducing the target protein level in comparison to *wasp* shRNA-1 (Fig. S1B). We therefore used this shRNA construct in our transfection experiment. In comparison to cells expressing GFP-Sh3px1 and the control shRNA, the cells expressing *wasp* shRNA-2 were unable to efficiently form protrusions (Fig. 7E,F,G. *P*=0.0018, unpaired *t*-test). It therefore appears that efficient protrusion formation by Sh3px1 requires Wasp activity.

We also attempted to determine whether protrusion formation by Sh3px1 required the actin nucleation factor, Scar. However, these results were difficult to interpret. Published findings, as well as our own experiments, indicate that Scar is required for spreading of S2 cells and lamellipodia formation (Fig. 2C,D) (Biyasheva et al., 2004; Kunda et al., 2003; Rogers et al., 2003). Thus, upon depleting Scar, a upstream defect in cell spreading precludes examining a potential role for Scar in Sh3px1-mediated protrusion formation.

## DISCUSSION

In this report, we have examined the consequence of depleting and over-expressing Sh3px1 in *Drosophila* S2 cells. Upon attaching to con A coated coverslips, S2 cells flatten and spread by forming circumferential lamellipodia (Rogers et al., 2003). Lamellipodia are actin rich structures that are often found at the leading edge of migratory cells. However, despite forming lamellipodia, S2 cells do not display directional motility. Within lamellipodia, F-actin is present in a dendritically branched organization (Svitkina and Borisy, 1999). This is in contrast to filopodia, in which actin filaments are organized as parallel bundles (Wood and Martin, 2002). Filopodia are rarely observed in S2 cells (Rogers et al., 2003).

S2 cells that were depleted of Sh3px1 were able to attach to con A coated coverslips but were deficient in lamellipodia formation (Fig. 2A,B). A similar phenotype was observed in cells depleted of the actin nucleation factor, Scar (Fig. 2C,D) (Biyasheva et al., 2004; Kunda et al., 2003; Rogers et al., 2003). We hypothesize that Sh3px1 functions in concert with Scar in forming lamellipodia. Consistent with this hypothesis, Scar was delocalized from the cortex in Sh3px-depleted cells, and Sh3px1 was delocalized in Scar-depleted cells (Fig. 2E-H). In addition, Sh3px1 has been shown to interact with Nck, a well-known regulator of Scar (Worby et al., 2001). An intriguing possibility is that Sh3px1 functions to coordinate signaling events at the plasma membrane with actin regulatory pathways that are required for forming lamellipodia.

Snx9, the human ortholog of Sh3px1, is known to dimerize and fold into a conformation that closely resembles a classical BAR domain (Pylypenko et al., 2007). Consistent with this finding, over-expression of full length Snx9, or the PXBAR domain, results in formation of membrane tubules (Pylypenko et al., 2007; Shin et al., 2008). Although the structure of Sh3px1 has not been solved, the primary sequence of its BAR domain is highly conserved with Snx9 (Fig. 4A). Thus, as expected, a small percent of cells over-expressing Sh3px1 formed tubules (Fig. 3B). Surprisingly, however, the majority of cells over-expressing Sh3px1 formed long protrusions (Fig. 3C-E).

How does over-expression of Sh3px1 result in the formation of protrusions? Answering this question is an important next step. The F-BAR protein Pacsin2 forms micro-spikes upon over-expression in mammalian cells (Shimada et al., 2010). In addition, the F-BAR domain of Nervous wreck (Nwk) has been shown to form long membrane protrusions upon over-expression in *Drosophila* S2 cells (Becalska et al., 2013). Although the precise mechanism of protrusion formation by these F-BAR proteins is not known, the authors have suggested a two-step mechanism. In the first step, the F-BAR protein binds to the membrane and generates a small invagination or tubule. In the next step, actin nucleators are recruited to this site, and local filament assembly drives protrusion formation (Becalska et al., 2013; Shimada et al., 2010).

Such a mechanism would also fit quite well for Sh3px1. We therefore tested whether F-actin was the driving force behind protrusion formation. Unfortunately, our results were inconclusive. On the one hand, protrusion formation by Sh3px1 was attenuated if the cells were simultaneously depleted of Wasp (Fig. 7E-G). This suggests a central role for actin in protrusion formation. However, visualization of F-actin using fluorescent Phalloidin indicated that whereas long protrusions were all positive for Phalloidin, some of the shorter protrusions displayed relatively little Phalloidin signal (Fig. 7A,B). We also used mRuby2-tagged Lifeact to visualize F-actin. Using this approach as well, we observed that some protrusions contained Lifeact, whereas others did not (Fig. 7C,D). One interpretation of these results is that protrusions might form independently of F-actin. However, a caveat with this interpretation is that a type of F-actin might be present at the tips of these protrusions that is not efficiently labeled using either Phalloidin or Lifeact.

Perhaps the best way to test the involvement of actin in protrusion formation would be to globally destabilize F-actin. Unfortunately, treating S2 cells with agents such as Cytochalasin D or Latrunculin A, resulted in the formation of ubiquitous cell protrusion by a different mechanism (Ling et al., 2004). When untransfected S2 cells, or cells transfected with GFP, are treated with these F-actin destabilizing drugs, they lose the cortical lamellipodia and unrestrained microtubule polymerization results in the formation of protrusions (Fig. S5) (Ling et al., 2004). We attempted to block these microtubule-based protrusions by co-treating S2 cells with actin and microtubule destabilizing drugs. However, this co-treatment resulted in excessive cell death (data not shown), thus complicating any mechanistic analysis. Therefore, due to technical reasons, we are not able to determine the precise role of F-actin in Sh3px1-mediated protrusion formation.

Our findings also suggest that Sh3px1 is not a passive player in forming tubules and protrusions. We identified specific residues within the PX and BAR domain that were critical for forming these membrane deformations. Thus, protrusion formation is not simply a consequence of aberrant actin polymerization. The ability of Sh3px1 to shape membranes appears to be just as important. Ultimately, we believe that the model proposed by Becalska et al., and Shimada et al., also applies to protrusion formation by Sh3px1 (Becalska et al., 2013; Shimada et al., 2010). It is difficult to imagine that Sh3px1 is capable of forming these long and dynamic protrusions without the aid of the underlying actin cytoskeleton. Conclusively proving this point, however, will require further experimentation.

In order to determine whether Sh3px1 possesses unique properties not shared by its mammalian orthologs, we over-expressed GFP-tagged versions of Snx9, Snx18 and Snx33 in S2 cells. This revealed differences between the mammalian proteins. Snx9 mostly formed long tubules, whereas Snx18 formed protrusions. Snx33 over-expression produced an intermediate phenotype consisting of short tubules and protrusions. In contrast to S2 cells, over-expression of Snx18 and Snx33 in Cos7 and HeLa cells respectively, resulted in tubule formation; protrusions were not observed (Dislich et al., 2011; Park et al., 2010). Thus, the type of membrane deformation induced by these proteins also depends on the cell type in which they are examined. S2 cells have a circumferential band of lamellipodia, whereas HeLa and Cos7 cells do not. Several factors, such as differences in organization of the actin cytoskeleton, composition of the plasma membrane, and expression and post translational modification of interacting partners, could all effect the type of membrane deformation that is produced by a BAR domain protein.

## MATERIALS AND METHODS

### Antibodies and staining reagents

The antibody against Sh3px1 was generated by cloning the cDNA corresponding to full-length Sh3px1 into the pGEX-2T vector. Expression of GST-Sh3px1 was induced by growing the cells at room temperature in 0.5 mM IPTG (Fisher). Protein lysates were prepared using the B-PER reagent (Pierce). The fusion protein was purified using sepharose beads coupled to glutathione (GE Healthcare Life Sciences). The purified protein was then injected into rabbits by Pacific Immunology. Specific antibodies against Sh3px1 were purified using a column containing Sh3px1 covalently attached to beads. Unless stated otherwise, the following corresponds to antibody dilutions used for immunofluorescence: Sh3px1 (1:200, 1:1000 western), Scar (1:100; P1C1-SCAR was deposited to the Developmental Studies Hybridoma Bank by S. Parkhurst), Ena (5G2 anti-enabled, 1:100, deposited to the Developmental Studies Hybridoma Bank by C. Goodman), Lamin DmO (clone ADL84.12, 1:1000 western, deposited to the Developmental Studies Hybridoma Bank by P.A. Fisher), Gamma tubulin (Sigma Aldrich, 1:1000 western), Alpha tubulin (Sigma Aldrich, 1:2000), GM130 Golgi marker (Abcam, 1:250), *Drosophila* Lamp1 (Abcam, 1:100), and anti-RFP (Chromotek, 1:1000 western). The following secondary antibodies from Life Technologies were used: Goat anti-rabbit 594 (1:400), Goat anti-rabbit 488 (1:200), Goat anti-mouse 594 (1:200), and Goat anti-mouse 488 (1:200). Goat anti-mouse HRP, anti-rabbit HRP and anti-rat HRP were used as secondary antibodies in western blotting (1:5000, Jackson Immunoresearch Laboratories). Aqua-Poly/Mount (Polysciences) was used as the anti-fade reagent.

F-actin was visualized using either Phalloidin-TRITC (Sigma Aldrich, 1:400) or Phalloidin-FITC (Sigma Aldrich, 1:200). DNA was stained using either DAPI (Sigma Aldrich). Cell membranes were visualized using either Cell Mask (Life technologies) or Fluorescein labeled *Lycopersicon esculentum* (tomato) lectin (1:100; Vector Laboratories).

### Cell culture

*Drosophila* S2 cells were obtained from Life Technologies and were grown in Schneider's media containing 10% Fetal Bovine Serum. In order to process cells for microscopy, S2 cells were spotted onto concanavalin A (Sigma Aldrich) coated coverslips as previously described (Rogers and Rogers, 2008). For live imaging, cells were spotted onto glass bottom coverslip dishes (MatTek). In order to globally knockdown Sh3px1, S2 cells were treated with either control dsRNAs targeting *gfp* or with dsRNAs targeting *sh3px1*. The cells were processed for immunofluorescence or western blot analysis four days after dsRNA treatment. The dsRNAs were prepared and administered to the cells as described previously (Rogers and Rogers, 2008). DNA was transfected into S2 cells using Effectene (Qiagen) following the directions provided by the manufacturer. The shRNA constructs were also transfected using Effectene.

### Immunofluorescence

Cells were fixed in 1× PBS containing 4% formaldehyde for 5 min. Subsequently, the cells were permeabilized for 5 min in 1× PBST (PBS containing 0.1% Triton X-100). In the case of fluorescent protein expression, the cells were then stained to visualize DNA or Actin, mounted onto slides, and imaged. In order to visualize the localization of non-fluorescent proteins, the coverslips were blocked with 1× PBST containing 5% normal goat serum (Life Technologies, referred to as blocking solution) for 30 min. Next, the coverslips were incubated with primary antibody diluted in the blocking solution. Cells were incubated with primary antibody for 1 h at room temperature. The coverslips were then washed three times with 1× PBST. Subsequently, the coverslips were incubated for 1 h at room temperature with secondary antibody diluted in the blocking solution. This was followed by four washes in 1× PBST. After the last wash, the cells were mounted onto slides and imaged.

### DNA constructs

The cDNAs for *sh3px1*, *wasp*, and the pARW vector were obtained from the *Drosophila* Genomics Resource Center. The cDNA for SNX9 was purchased from Open Biosystems. The cDNA construct for mouse SNX18 was made by gene synthesis (Genewiz). The cDNA for SNX33 was cloned from total RNA prepared for P3.5 mouse brain. The cDNA for wasp was cloned into the pARW vector by Gateway cloning (Life Technologies). In order to over-express constructs in S2 cells, cDNAs corresponding to GFP, *sh3px1,* SNX9, SNX18 and SXN33 were cloned into the pUASp-attB-K10 vector (Koch et al., 2009). Expression of the tagged proteins was induced by co-transfection with a plasmid expressing Gal4 driven by the Actin5C promoter (gift of Jocelyn McDonald, Kansas State University). TagRFPt versions of Snx9 and Sh3px1 were made by first obtaining a plasmid encoding TagRFPt-EEA1 from Addgene (Addgene plasmid # 42635; donated by Silvia Corvera; Navaroli et al., 2012). The TagRFPt sequence from this plasmid was then cloned into pUASp-attB-K10 (Koch et al., 2009). The GFP tagged F-BAR domains of Cip4, Nwk and Mim, as well as the mCherry tagged F-BAR of Syndapin were kindly provided by Avital Rodal (Becalska et al., 2013). In order to visualize F-actin in live cells, a plasmid expressing mRuby2-Lifeact was obtained from Addgene (Addgene plasmid # 54674; donated by Michael Davidson; Lam et al., 2012). The sequence for mRuby2-Lifeact was subsequently cloned into pUASp-attB-K10. For knock-down studies, shRNAs targeting the desired gene were constructed as previously described (Ni et al., 2011). The shRNA oligoes were cloned into the Valium 20 vector (obtained from the DF/HCC Plasmid Resource Core at Harvard University). The following shRNA targeting sequences were used. *sh3px1* shRNA1 (5′-ACCAGTGACTACGATAACAAA-3′); *wasp* shRNA-1 (5′-TCGGTGAAGCAATACAGTGTA-3′); *wasp* shRNA-2 (5′-CGGGGTGGTGTGCTTCGTCAA-3′), and *scar* shRNA (5′-CTCGACAAGCTTAATGTCTAT-3′). The point mutations in *sh3px1* were generated using the Q5 site-directed mutagenesis kit (NEB).

### Lysates and western blotting

Soluble lysates were prepared by resuspending S2 cell pellets in RIPA buffer (50 mM Tris.Cl pH 7.5, 150 mM NaCl, 1% NP-40, 1 mM EDTA) containing protease inhibitors (Halt protease inhibitor cocktail kit, Pierce). The cell suspension was then passed several times through a 25 gauge needle. Lysates were cleared by centrifugation at 10,000 ***g*** for 5 min at 4°C. 50 μg of each lysate was run on a precast TGX gel (BioRad), transferred to nitrocellulose, and probed with the indicated antibodies. The chemiluminescent signal was captured using a UVP bioimaging system.

### Drug treatments

In order to destabilize actin filaments, cells were treated with 1 µM Latrunculin A (Life Technologies and Santa Cruz Biotechnology). In order to destabilize microtubules, cells were treated with 2 μg/ml Colcemid (Sigma Aldrich). Both drug treatments were performed for 2 h.

### Microscopy

Fixed samples were imaged on a Leica 510 upright confocal microscope or on a Leica 780 upright confocal microscope. For the live imaging experiments, cells adhered to No.0 glass bottom dishes (MatTek) were imaged on a Leica 780 inverted confocal microscope. Fixed and live images were processed and analyzed using the open source software, Fiji. Quantification of co-localization (Pearson's coefficient) was done using the Zen software on the Leica 780 upright confocal microscope. All imaging experiments were performed at the Georgia Regents University Cell Imaging Core Laboratory.
